# Towards interpretable speech biomarkers: exploring MFCCs

**DOI:** 10.1038/s41598-023-49352-2

**Published:** 2023-12-21

**Authors:** Brian Tracey, Dmitri Volfson, James Glass, R’mani Haulcy, Melissa Kostrzebski, Jamie Adams, Tairmae Kangarloo, Amy Brodtmann, E. Ray Dorsey, Adam Vogel

**Affiliations:** 1 Takeda Pharamaceuticals, Data Science Institute, Cambridge, MA 02142 USA; 2 Center for Health + Technology (CHeT), University of Rochester Medical Center, Rochester, NY USA; 3 Massachusetts Institute of Technology, CSAIL, Cambridge, MA 02139 USA; 4 Monash University, Melbourne, VIC Australia; 5 University of Melbourne, Parkville, VIC 3010 Australia; 6 Redenlab Inc, Melbourne, VIC 3010 Australia

**Keywords:** Biomarkers, Dementia, Parkinson's disease

## Abstract

While speech biomarkers of disease have attracted increased interest in recent years, a challenge is that features derived from signal processing or machine learning approaches may lack clinical interpretability. As an example, Mel frequency cepstral coefficients (MFCCs) have been identified in several studies as a useful marker of disease, but are regarded as uninterpretable. Here we explore correlations between MFCC coefficients and more interpretable speech biomarkers. In particular we quantify the MFCC2 endpoint, which can be interpreted as a weighted ratio of low- to high-frequency energy, a concept which has been previously linked to disease-induced voice changes. By exploring MFCC2 in several datasets, we show how its sensitivity to disease can be increased by adjusting computation parameters.

## Introduction

The last decade has seen an increase in the use of speech for health monitoring, with a focus on studies in neurological^1,2^ and respiratory disease^3,4^. This is in part driven by increased ease in recording good-quality data using either smartphones^5^ or cloud-based platforms^6^. Analysis of this data has used a mix of interpretable endpoints (prosodic measures related to timing and pitch, etc.) as well as speech parameterizations originally developed for speech recognition. This latter category of parameterizations (which includes MFCCs, or Mel Frequency Cepstral coefficients^1^, RASTA coefficients , and deep learning derived embeddings^7^) often leads to high performance in classification or regression tasks, but low interpretability. This lack of interpretability makes it difficult to link acoustic features to disease biology and diminishes their utility to clinicians and patients.

MFCCs were originally developed for speech recognition^8^ and have found diverse use as voice descriptors, for example in emotions recognition^9^ or speech disorder classification^10^. Among MFCC features, the second MFCC coefficient (MFCC2) has been identified as a valuable feature for distinguishing phonation of healthy subjects from people with Parkinson's disease  (PD)^11–13^ or other diseases such as major depressive disorder^14^ and Alzheimer’s disease^15^.

Speech changes caused by PD are the result of deficits across multiple subsystems of production, including respiration, phonation, articulation, resonance, and prosody^16^. Relevant to MFCCs, respiration is impacted by reduced airflow volume during speech and increased vital capacity percentage per syllable; phonatory deficits result in reduced loudness, and breathy and harsh voice quality; articulatory deficits are characterized by imprecise production of consonants and distorted vowels; and reduced velopharyngeal control manifests in hypernasal resonance. The combination of these changes to speech appears to be reflected in MFCC values, which are thought to model irregular movements in the vocal tract^17^. MFCC features have been shown in multiple studies to significantly differentiate PD subjects from controls, whether used alone^18^ or in combination with other features^19,20^. Irregular movements can be the result of changes in respiratory support and pressure, altered vocal fold dynamics, or impaired articulator movement-all consequences of PD.

Fronto-temporal dementia (FTD) is another example of a neurodegenerative disease that results in complex speech deficits^21^. There are four variants within the FTD spectrum including the behavioral variant of FTD (bvFTD) and three primary progressive PPA syndromes: nonfluent/agrammatic (nfvPPA), semantic (svPPA), and logopenic (lvPPA). Each present with a distinct communication phenotype, resulting from a combination of cognitive-linguistic and motor impairments. bvFTD has documented speech changes across tasks^2^ including reduced rate and accuracy on alternating syllable production tasks. The non-fluent variant yields stark motor speech changes due to apraxia of speech^22^. Errors in vowel and consonant production are common in nfvPPA and lvPPA, but the underlying mechanisms leading to these changes are different. Imprecise production of consonants and vowel distortion in nfvPPA are thought to be the result of impaired motor planning (apraxia) and in some cases a concomitant motor planning deficit (dysarthria). Deficits in sound accuracy in lvPPA are considered a consequence of underlying phonological representation and retrieval, and therefore related to language function. The semantic variant of PPA is largely characterized by word finding deficits and is less associated with frank motor speech impairments.

The discrete speech phenotypes in PD and FTD offer an opportunity to explore the differential impact of disease on commonly used and potentially useful objective markers of speech. As described, MFCCs provide information on vocal tract dynamics, which change based on pathology and manifestations of the disease. The underlying mechanisms driving speech profiles in PD and FTD are different. These contrasts may help explain why we see different MFCC values across diseases and can contribute to our understanding of the metric itself. Data on MFCCs in disease may also build a stronger evidence base for their use in clinical trials and for monitoring disease in the future.

With this motivation, we explored MFCC features (and MFCC2 in particular) in several datasets in PD, frontotemporal dementia (FTD), and healthy speakers. We demonstrate that a) by tuning the MFCC2 calculation to include more high frequencies, we can affect its performance, and b) MFCC2 appears to depend strongly on sex but not age. Finally, we explore correlations between MFCCs (including higher-order MFCCs) and more interpretable voice descriptors.

###  MFCC computation and interpretation

MFCC2 can be interpreted as a weighted ratio of low- to high-frequency energy, as outlined in the following paragraph. This relationship has been previously noted in the literature^23^ although many papers use MFCC features as black-box features. The Discussion reviews existing literature which links low-to-high frequency energy ratios to voice pathology in PD as well as other diseases.

**Figure 1:**
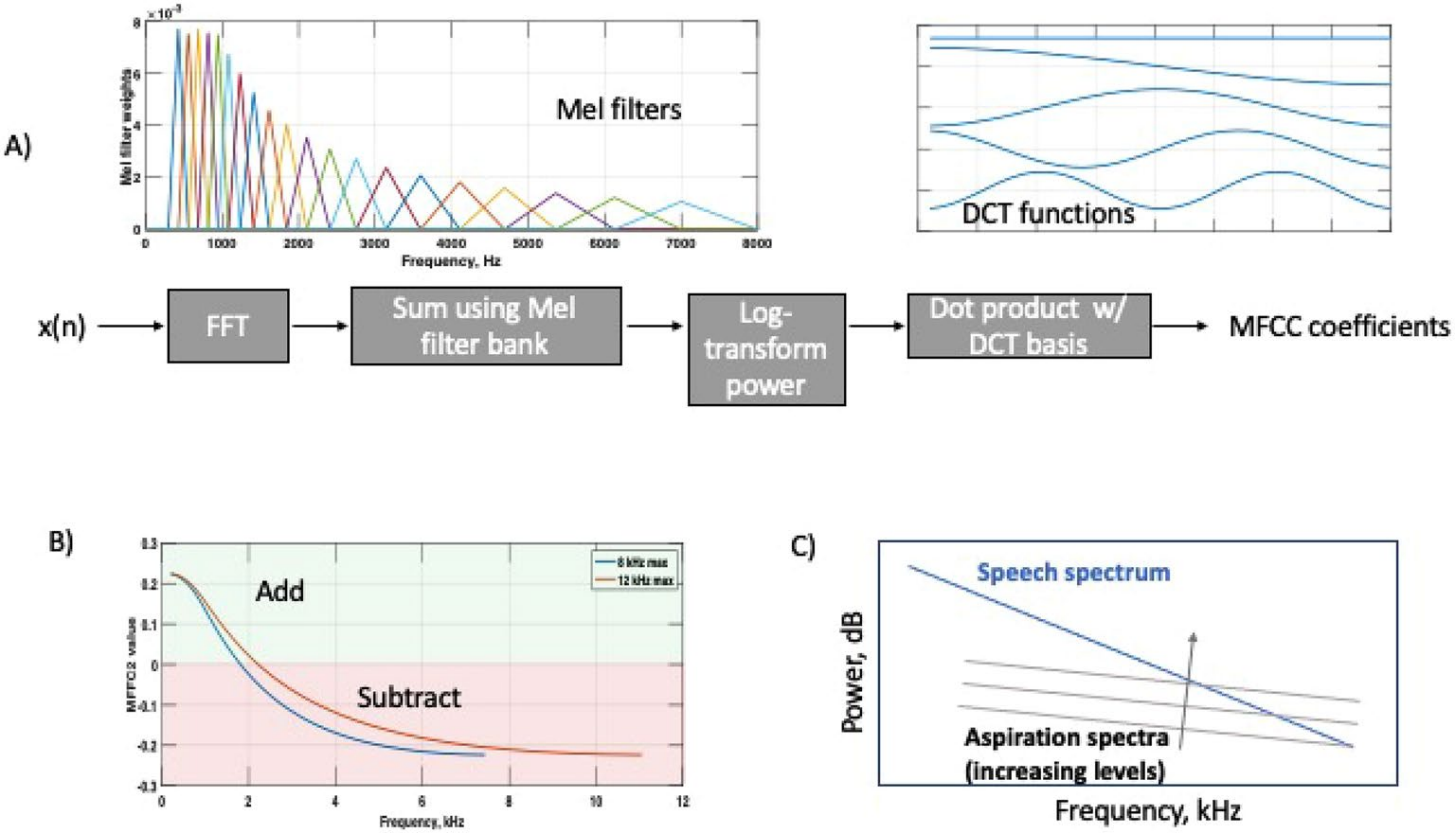
Overview of MFCC calculation; (**A**) shows a schematic of the MFCC computation; (**B**) shows the MFCC2 cosine term mapped to frequency in Hz, for maximum frequency values of 8 and 12 kHz; (**C**) shows a ‘cartoon’ view illustrating how increasing aspiration or other noise can impact affect the overall spectrum at high frequencies.

Figure 1A briefly summarizes the MFCC calculation. The input signal is first transformed to create a spectrogram. Mel frequency filters are then applied to resample the frequency axis in a manner that mimics the roughly logarithmic pitch sensitivity of human hearing, with finer resolution at lower frequencies and coarser resolution at high frequencies. The Mel-filtered data are then log-transformed and processed with a cepstral transform, which amounts to multiplying the Mel spectra by a series of cosine terms. As shown in Fig, 1A), MFCC1 is a constant feature capturing overall energy, MFCC2 is a half-cycle of cosine, etc. The MFCC coefficients are computed by multiplying the log-transformed Mel spectra by the cosine terms and then summing across frequency.

Figure 1B shows the cosine term associated with MFCC2, remapped from Mel frequency back to actual frequency in Hz. This figure indicates that MFCC2 is adding a weighted sum of low frequency log(energy) and subtracting off a weighted sum of high frequency log(energy). As $log(a)-log(b) = log(a/b)$, MFCC2 can be interpreted as a form of low-to-high frequency energy ratio, with the lowest and highest frequencies contributing most strongly due to the weighting applied.

Figure 1B also shows two MFCC2 curves. MFCC2 computation requires the user to specify the maximum frequency used in calculation. While 8 kHz is a common upper limit, we show below that increasing the maximum frequency can be beneficial. Figure 1C shows how low and high frequency spectra can be differentially affected by additive phonation-related noise, as will be discussed in detail below.

## Results

We examined sustained phonation recordings from three datasets, with patient characteristics shown in Table 1. All three datasets contain control participants. The University of Melbourne/Monash dataset also includes participants with FTD (32 behavioral variant , 11 semantic variant, and 12 nonfluent variant), while the WatchPD dataset includes participants with PD. The PD participants were recruited soon after diagnosis so have fewer years of disease than the FTD participants. Each subject listed in Table 1 provided a single phonation recording. For the Melbourne dataset, a small number of participants made more than one clinic visit, so Tables 2, 3 include only the first visit for each subject.

**Table 1:** Subject information.

Dataset	Diagnosis	Age	Years since diagnosis	# Participants (male/female/other)
Melbourne	FTD	65.0 (60.2/71.0)	3 (2/5.5)	36/19/0
Melbourne	Controls	63.0 (56.0/70.0)	–	50/56/0
WatchPD	PD	66.0 (55.0/71.0)	<1	29/25/0
WatchPD	Controls	61.0 (54.5/69.5)	–	19/24/0
CLAC	Controls	33.0 (27.0/42.0)	–	799/800/11

Age and Years since Diagnosis are listed as median (25th percentile/75th percentile).

**Figure 2:**
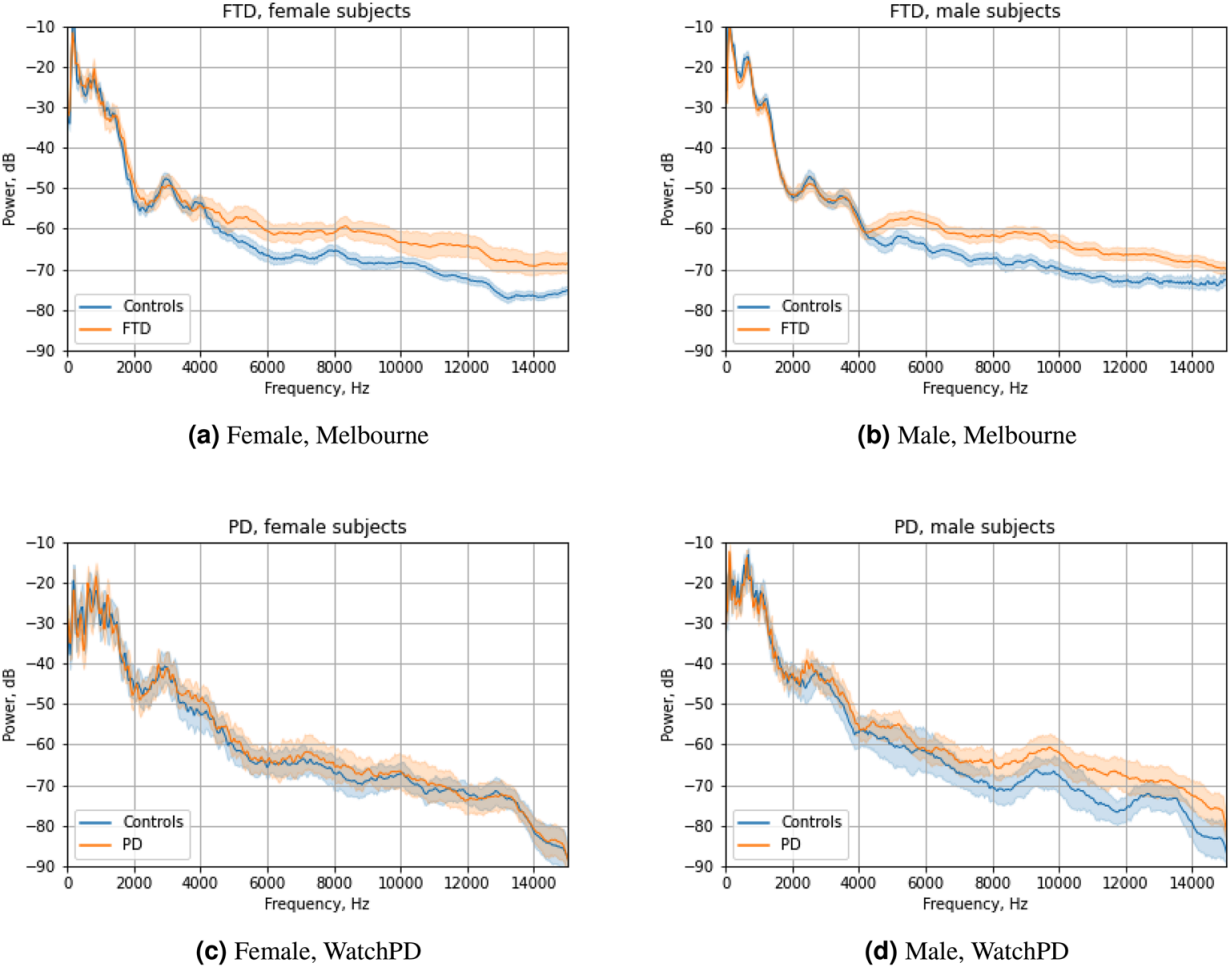
Mean spectra (with 95th percentile confidence limits) for Melbourne and WatchPD datasets. Note the generally higher values above 4 kHz for non-healthy participants.

Figure 2 shows the averaged acoustic spectra (showing mean and 95th percentile confidence limits for the mean) for the Melbourne and WatchPD datasets, comparing controls to participants with neurological disease (FTD or PD) (called “cases” below). Because spectral characteristics vary by sex, plots are shown separately for males and females. In general, these plots indicate that FTD/PD participants have higher acoustic power at high frequencies as compared to controls. This is seemingly more evident in men, as well as in the FTD participants, who have greater years of disease duration.

We next computed various low-to-high ratios for our datasets. MFCC2 was computed with the standard 8 kHz maximum frequency, as well as MFCC2 with a 12 kHz maximum frequency. Following Hillenbrand and Houde^24^, we compare energy above and below 4 kHz using an Energy Ratio metric; while they computed a high-to-low ratio, we instead compute a low-to-high ratio for easier comparison to MFCC2 (see Methods). Table 2 lists AUC (area under the curve) values for these metrics from ROC curves for Melbourne and WatchPD datasets (no ROC curves are shown for CLAC as the database only contained control participants). MFCC2 with 12 kHz maximum frequency has the best average AUC, with the energy ratio has the lowest. However, confidence intervals are overlapping, so it is not possible to conclude any particular metric is statistically superior. Descriptive statistics for these metrics are shown in Table 3. Note that in all cases, control participants have higher mean values for all three metrics.

**Table 2:** ROC Area under the curve (AUC) for different metrics and datasets; mean AUC and 95th percentile confidence intervals are shown.

Metric	Dataset	AUC, Female	AUC, Male
MFCC2, 12kHz	Melbourne	**0.82 (0.66–0.97)**	**0.78 (0.67–0.89)**
MFCC2, 8kHz	Melbourne	0.69 (0.51–0.86)	0.73 (0.61–0.86)
Energy Ratio	Melbourne	0.73 (0.58–0.87)	0.75 (0.64–0.87)
MFCC2, 12kHz	WatchPD	**0.61 (0.45–0.77)**	**0.69 (0.54–0.84)**
MFCC2, 8kHz	WatchPD	**0.61 (0.45–0.77)**	0.67 (0.51–0.83)
Energy Ratio	WatchPD	0.59 (0.43–0.75)	0.58 (0.41–0.75)

Bold values highlight the metric with the highest average AUC for each dataset.

**Table 3:** Descriptive statistics for MFCC2-related metrics, by dataset, sex and diagnosis (SD denotes standard deviation).

Dataset	Gender	Diagnosis	MFCC2, 12 kHz		MFCC2, 8 kHz		Energy ratio	
			Mean	SD	Mean	SD	Mean	SD
WatchPD	Female	Healthy	136.0	22.3	95.8	19.1	33.5	6.8
WatchPD	Female	PD	128.1	21.3	89.6	16.0	31.1	6.8
WatchPD	Male	Healthy	148.8	20.6	107.4	15.7	34.9	8.1
WatchPD	Male	PD	133.2	20.4	98.3	14.5	32.3	6.6
Melbourne	Female	Healthy	143.4	11.8	109.9	10.7	38.3	4.7
Melbourne	Female	FTD	126.8	18.8	101.5	13.0	34.5	4.1
Melbourne	Male	Healthy	148.4	13.1	112.5	8.5	38.8	4.1
Melbourne	Male	FTD	131.9	14.9	103.5	10.4	34.7	4.5
CLAC	Female	Healthy	131.1	29.2	90.8	24.4	30.1	9.1
CLAC	Male	Healthy	137.1	29.9	96.0	23.8	30.4	9.8
CLAC	Other	Healthy	123.2	30.6	85.1	30.2	27.6	9.9

**Figure 3:**
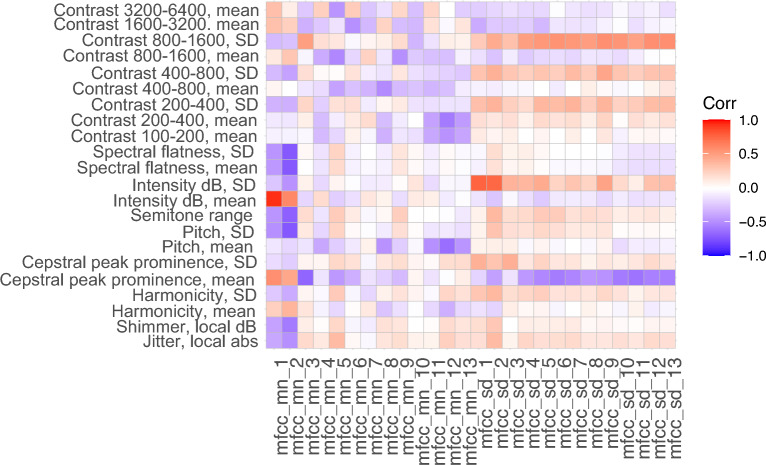
Pearson correlation coefficients between MFCC features and non-MFCC features (Melbourne data), for non-MFCC features that have at least minimal (> 0.3 absolute value) correlation with at least one MFCC feature. Spectral contrast features are denoted ’Contrast’ with associated frequency ranges; SD denotes standard deviation.

Next, we analyzed higher-order MFCC coefficients to understand how they map onto more interpretable features. Thus, Fig. 3 shows computed Spearman correlations between MFCC features (mean value and standard deviation across each vocalization) and more interpretable speech features, such as spectral contrast in various frequency ranges^25^, spectral flatness^25^, signal intensity metrics, pitch metrics, and several measures of voice quality (cepstral peak prominence, jitter, shimmer)^26,27^, for the Melbourne dataset. The x-axis shows the derived MFCC metrics; the y-axis only includes metrics which showed at least low correlation (>0.3 absolute value) correlation with at least one MFCC metric. Note that multiple features correlate to MFCC2, and that measures of variability (for example standard deviations of spectral contrasts or signal intensity) correlate to MFCC standard deviation metrics.

### Statistical results

**Figure 4:**
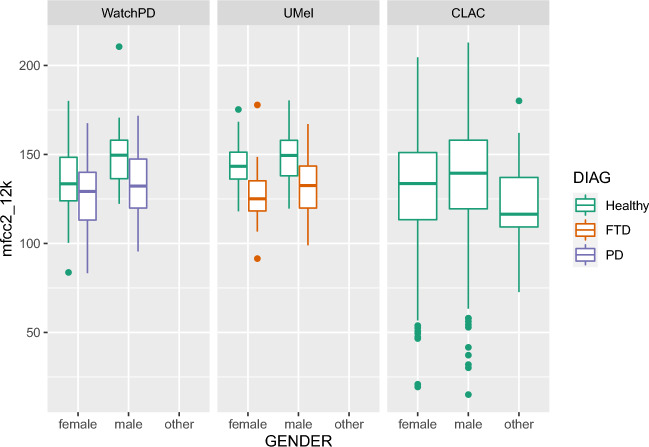
MFCC2 (fmax = 12 kHz) characteristics, showing dependence on gender and diagnosis. Detailed analysis found in the Statistical Analysis section.

We first explored whether MFCC2 and related metrics were dependent on sex, age and dataset, as well as diagnosis. We modeled each MFCC2 endpoint as a linear combination of factors using the regression function lm in R version 4.2.2. A model selection process using the Akaike Information Criterion (AIC) led to selection of a model which includes gender, dataset, and age. Because dataset and diagnosis are confounded, this analysis was done for control participants only. For MFCC2 with fmax = 12 kHz, there was a highly significant effect of gender (males were higher, *p* <0.001) and a significant effect of dataset (CLAC values were lower, *p* <0.05) with no significant effect of age. The corresponding boxplots for MFCC2 with fmax = 12 kHz are shown in Fig. 4 for the different datasets, by gender and diagnosis. MFCC2 values are higher in men, reflecting the increased low-frequency content in these speakers (and are lower in disease, reflecting the increased high-frequency noise seen in Fig. 2). For MFCC2 with fmax = 8 kHz (not plotted), these findings were repeated, but also there were also significant effects of age (values decreased with a small slope of roughly 1 point per decade, p<0.05) and also Melbourne values were significantly higher than WatchPD values. Within FTD subjects, we performed ANOVA analysis (after verifying normality assumptions were met) and found no significant differences between FTD subtypes on MFCC2 metrics, though it is important to note our sample sizes are small. Moderate to good correlations were found in each of the three datasets between MFCC2 and the Energy Ratio metric described above (Pearson correlations are 0.82 in the WatchPD data, 0.60 in the Melbourne data, and 0.74 in CLAC).

## Discussion

In this section we first discuss findings for the MFCC2 feature, followed by observations about higher-order MFCCs.

The acoustic spectra in Fig. 2 show that (especially in the FTD cohort) differences between cases and controls are small at lower frequencies but are noticeable above roughly 4 kHz. As the MFCC2 can be interpreted as a low-to-high energy ratio, the metric appears to be exploiting this spectral difference to discriminate the presence of disease.

There is a well-established literature that links low-to-high energy ratio differences to voice distortion. Breathy voice can be characterized in the low-frequency range via increased amplitude of the first harmonic, as the glottal waveform becomes more rounded due to non-simultaneous closure along the length of the vocal cords^24^. More relevant to MFCC2, high-frequency energy also increases due to the presence of turbulent airflow associated with breathy voice. Hillenbrand and Houde^24^ note, “.. the presence of aspiration noise, which is stronger in the mid and high frequencies than in the lows, can result in a voice signal that is richer in high-frequency energy than nonbreathy signals.” As illustrated in a ‘cartoon’ view in Fig. 1C, aspiration noise, generated by turbulent flow, tends to fall off less rapidly with frequency than voice. As aspiration noise increases, it primarily impacts the high-frequency part of the overall (voice plus aspiration noise) spectrum but remains negligible compared to voice at the low frequencies. Hillenbrand and Houde thus proposed a high-to-low (H/L) ratio, comparing average energy above 4 kHz to average energy below 4 kHz, to capture breathy voice aspiration noise. Given the high correlation between MFCC2 and this high-low ratio (see Fig. 4b), one also would expect that MFCC2 would capture breathiness and aspiration noise.

Links between disease and low-high frequency ratio have been established, especially in PD (we are not aware of similar studies in FTD). As reviewed by Ma *et al.*^28^, multiple studies using laryngoscopic and stroboscopic data have showed that PD is often associated with incomplete glottal closure, asymmetric vocal fold closure, and slower glottal opening, with many of these changes likely due in increased muscle rigidity or poor motor control. Air leakage due to incomplete glottal closure has acoustic effects including increased breathiness and reduced harmonics-to-noise ratio^29^, as additional high frequency noise is present. Incomplete glottal closure can also lead to reduced loudness (hypophonia) due to lower pressure and reduced breath support. The acoustic impacts of incomplete glottal closure are reported to include increased jitter and reduced HNR, also linked to breathiness^16,28^.

It is important to note that changes in low-to-high energy ratio differences are not disease-specific. Thus, similar ratios splitting high and low frequencies appear sensitive to changes in speech and voice resulting from Huntington’s disease^30^, illicit drug use^31^, congestion^32^, and fatigue^33^.

Our data shows a clear impact of gender on MFCC2. In healthy participants, MFCC2 values are higher in men than women in all cohorts (Table 3), presumably reflecting the fact that male voices are skewed to lower frequencies.

Our results also show that technical issues and computation settings may affect the utility of MFCC2 as a clinical marker. Figure 2 suggests that differences between cases and controls become larger at higher frequencies. The highest upper bound we considered for MFCC computations in this work was 12 kHz. In principle, further increasing the upper range might improve separability between groups. However, manual examination of our data shows occasional high-frequency noise of undetermined source at frequencies above roughly 12-15 kHz. Thus, we limited the upper frequency range to 12 kHz in our calculation. This upper limit is based on engineering judgement, not extensive data exploration. If phonations were recorded in a very quiet environment using high quality hardware^34^, it might be beneficial to include higher frequencies.

Because MFCC2 values depend on the frequency limits used in computation, it is important that MFCC parameter settings should be reported along with findings, to better allow comparisons between different studies. Widely used MFCC implementations such as librosa^25^ default to using half the sampling rate as the upper limit, which means that MFCC values could easily vary depending on recording settings (and may include very high-frequency noise, as noted above).

While our focus here is on MFCC2, we also explored higher-order MFCCs. Just as MFCC2 is interpreted as a low-to-high energy comparison, it would be possible to interpret MFCC3 as a mid-frequency to (low+high)-frequency ratio, MFCC4 as a ratio of two frequency bands to two slightly higher frequency bands, etc. (see the cosine shapes in Fig. 1a), with higher-order MFCCs sampling faster variation across the frequency spectrum. However, the physical / biological meaning of such ratios becomes increasingly unclear as the MFCC coefficient number increases.

We calculated alternate and potentially more intuitive metrics for capturing structure of the acoustic spectra; thus we computed spectral contrast^25^ (which measures spectral amplitude peak-to-trough within selected frequency bands), spectral flatness (which measures overall peakiness of the spectrum, with high values representing flat spectra and low values representing tonal-dominate spectra), as well more standard speech features (described in Methods; see also^26^). Figure 3 shows how the full set of MFCC coefficients correlates to these features, after dropping features with minimal correlation to MFCCs. Several observed correlations are expected; for example, average signal intensities as measured by mean values of MFCC1 and RMS signal intensity are highly correlated. Also, decreased stability of voice intensity is positively correlated with MFCC SDs, as is increased SD of spectral contrast features. More interestingly, increasing voice clarity (as captured by mean values of Cepstral Peak Prominence, or CPP) is negatively correlated to MFCC SDs, suggesting clearer voices also have more stable spectral structure over the phonation. Instability in voice clarity (measured by the SD in cepstral peak prominence or harmonicity) correlates with increased MFCC SD. Instability of the spectral structure as captured by MFCC SD and spectral contrast SD appears correlated; spectral contrast SD potentially is more interpretable in that the frequency bands contributing to instability are identified.

Figure 3 shows several interesting correlations to MFCC2 (mfcc_mn_2). MFCC2 decreases (moves in the direction of pathology) when frequency variability increases, as measured by jitter, pitch SD, and pitch semitone range. MFCC2 also decreases when amplitude variability increases (as measured by shimmer or intensity variability). MFCC2 is higher when the spectrum is more dominated by tonal components (increasing harmonicity or CPP, or decreasing spectral flatness).

### Limitations

While we characterized MFCC across several datasets, several of these datasets were relatively small, which limits conclusions that can be drawn. For example, larger datasets would be helpful to characterize potential sex-specific changes in MFCC2 with disease. Table 2 shows that MFCC2 better discriminates Parkinson’s in men (higher AUC). It has been previously established that PD differentially affects male and female voices^1,7^. A possible physical explanation is that male voices containing relatively less mid-to-high frequency energy would be more affected by addition of aspiration noise in this frequency range (see Fig. 1C). However, the same pattern is not observed in FTD participants,which may be related to the small size of the FTD cohort. The small size of our FTD cohort also impacts our ability to perform differential analysis of subtypes. In both cases the uncertainty in the AUCs (seen in the confidence bounds) argues for repeating these analyses in additional datasets.

We also observed differences between datasets (for example greater variability in the normative CLAC dataset) which may be impacted by technical aspects of the recording. The datasets use different recording setups; Melbourne is a mixture of in-lab recordings and at-home (smartphone) recordings, WatchPD consists of in-clinic recordings made with an iPhone (note that only data from the WatchPD baseline visit are analyzed here; the complete WatchPD dataset also includes at-home recordings), and CLAC was recorded via internet browsers using Amazon Mechanical Turk. This means CLAC data are subject to data compression artifacts that may be variable depending on browser, service provider, etc. Manual review uncovered CLAC recordings in which the sustained phonation was distorted after the first second or so, perhaps because noise cancellation algorithms incorrectly identify the sustained phonation as a form of background noise (this issue impacts sustained phonation more than regular speech, as algorithms presumably target hum-like signals). These variations in browser processing may explain the noticeably higher standard deviations seen in metrics computed from CLAC (Table 3). This suggests that the acoustic quality of internet-acquired data be carefully reviewed, and that ideally browser settings be controlled to disable processing algorithms, especially when subtle acoustic features of speech are being analyzed.

Given the difficulty in interpreting higher-order MFCCs, interpretable alternative metrics for capturing spectral structure would be of value. As a first step, we performed an initial study of spectral contrast, spectral flatness, and other features, showing correlations to MFCC coefficients (Fig. 3). However, further exploration of alternative metrics would be of value.

## Methods

We analyzed recordings of sustained vowel phonation (“aaah”) from baseline clinic visits in the WatchPD study^5^, and from data collected at the University of Melbourne (consisting of healthy elderly controls^35^ and FTD participants^2^). We also utilize the public-domain CLAC dataset^6^ of normative speakers, collected using Amazon Mechanical Turk.

In each dataset, recordings were first automatically segmented using custom Python code to identify the vowel phonation. Processing first detected voiced regions of speech using the voicing/pitch detection from Parselmouth^27^. The initial 0.75 s of voiced data were discarded to remove transient effects, and the next 2.5 s were retained for analysis. If no segment was detected, the recording was not analyzed (and is not included in Tables above). The segmented waveform was then processed using Librosa^25^ to compute MFCC coefficients, using 133 Hz as a lower bound and either 8 or 12 kHz as upper bounds. Both mean and standard deviation MFCC features were computed across all frames in each phonation. In addition, the acoustic spectra were computed using the scipy-signal implementation of the Welch periodogram method, using 20 ms, 50% overlapped Hanning windows, as plotted in Fig. 2. These (linear) spectra $P_{xx}(f)$ were used to compute the Energy Ratio metric:

1 $$\begin{aligned} ER, dB = 10 \log _{10} \left( \frac{\int _0^{4000} P_{xx}(f) df }{\int _{4000}^{f_{max}} P_{xx}(f) df} \right) \end{aligned}$$

where $f_{max}=12,000$ Hz. Note that whereas Hillenbrand and Houde^24^ formed a ratio of high-to-low energy, we compute low-to-high for easier comparison with MFCC2.

Additional (non-MFCC) metrics were computed from the segmented phonation recordings. The Parselmouth-Praat interface^27^ was used to compute several speech clarity metrics, including cepstral peak prominence (CPP), harmonicity, jitter (‘localabs’ variant) and shimmer (‘local dB’ variant). The same package was used to compute pitch metrics (mean pitch, standard deviation of pitch, and semitone range). The librosa package was used to explore spectral features, to explore spectral descriptors that are more interpretable than higher-order MFCCs; thus spectral contrast was computed (default settings) as well as spectral contrast in octave bands, starting at 100 Hz (so, 100–200 Hz, 200–400 Hz, etc. up to 3200–6400 Hz).

Statistical analysis was performed in R version 4.1.0. AUC analysis was performed using the pROC package (version 1.18.0) which uses bootstrapping to estimate confidence intervals. For AUCs shown in Table 3, MFCC2 was the only feature; thus no classifier is required, as MFCC2 can be used as a scalar test statistic. AUC results in Table 3 were generated by sweeping the threshold values across the range of MFCC2.

## Data Availability

Raw audio for the CLAC dataset is available at https://groups.csail.mit.edu/sls/downloads/clac/. The extracted features for the CLAC dataset are available at https://github.com/brianhtracey/mfcc2_related. Extracted features for other datasets may be available upon reasonable request.

## Code availability

Core python code for MFCC2 feature extraction is available at https://github.com/brianhtracey/mfcc2_related.
